# The impact of cancer prevention education on the mental health of college students based on the difference-in-differences method

**DOI:** 10.3389/fpubh.2024.1446225

**Published:** 2024-10-08

**Authors:** Li Jia, Qian Du, Qian Huang, Yawen Pang

**Affiliations:** ^1^School of Humanities and Management, Guangdong Medical University, Dongguan, China; ^2^School of Humanities, Taiwan National Chi Nan University, Taiwan, China

**Keywords:** cancer prevention education, mental health, college students, difference-in-differences, disease awareness, psychological resilience

## Abstract

**Background and objective:**

Cancer, as the second leading cause of death worldwide, poses significant challenges to human health and socio-economic development. In recent years, the incidence of cancer has shown a trend toward younger populations, drawing attention to cancer prevention education among college students. However, research on the specific impact of cancer prevention education on the mental health of college students is limited. This study aims to explore the impact of cancer prevention education on the mental health of college students, revealing the mediating role of disease awareness and the moderating roles of psychological resilience and cultural differences.

**Methods:**

A difference-in-differences (DID) approach was used, involving 1,670 freshmen from a Chinese university, divided into an experimental group (*n* = 835) and a control group (*n* = 835). The experimental group received a semester-long cancer prevention education program. Data were collected monthly from November 2022 to June 2023 using the Depression Anxiety Stress Scales (DASS-21) and a custom Disease Awareness Scale.

**Results:**

The study found a significant improvement in mental health scores among the experimental group, with an average increase of 14.738 points on the DASS-21 scale (*p* < 0.001), representing a 23% reduction in stress, anxiety, and depression levels compared to the control group. Disease awareness in the experimental group improved by 17%, as measured by the Disease Awareness Scale, with a mediation effect of 3.563 points (*p* < 0.001). Furthermore, psychological resilience and cultural differences moderated the impact of the education program, with those scoring higher in resilience showing an additional 8% improvement in mental health scores (moderation effect = 0.892, *p* < 0.001), and cultural differences accounting for a 5% variance (moderation effect = 0.756, *p* < 0.001) in the outcomes.

**Conclusion:**

This study demonstrates that systematic and scientific cancer prevention education has a significant positive impact on the mental health of college students. Universities should promote comprehensive and personalized health education strategies to improve disease awareness, foster psychological resilience, and emphasize cultural differences, thereby enhancing the overall physical and mental health of college students and promoting their holistic development. This finding provides important empirical support and theoretical basis for the design and implementation of health education in universities.

## 1 Introduction

Cancer imposes a significant clinical burden, disrupts normal social order, and drains many economic resource (1). According to data released by the International Agency for Research on Cancer (IARC) of the World Health Organization, there were 20 million new cancer cases globally in 2022, with nearly 9.7 million deaths. In China alone, there were approximately 4.8 million new cancer cases (accounting for 24% of the global total) and around 2.6 million cancer deaths (accounting for 26.7% of the global total) in 2022 (2). Despite improvements in the survival rates of many cancer patients, cancer remains the second leading cause of death worldwide and is projected to surpass heart disease as the leading cause of death within the next 40 years (3, 4). Notably, young cancer patients tend to be overlooked compared to the oder adult. In recent years, the incidence of cancer has been rising among young people (3, 5). Effectively preventing cancer and reducing cancer risk among young people has become an urgent public health issue. College students represent a crucial demographic in the fight against cancer due to their high learning capacity and potential for long-term behavior modification. However, they also face unique challenges related to cancer risk behaviors. Recent studies indicate that a significant proportion of college students engage in behaviors that increase their cancer risk (5, 6), such as smoking, excessive alcohol consumption, unhealthy dietary habits, and physical inactivity (7–9). These behaviors are often exacerbated by the newfound independence and lifestyle changes associated with university life. Additionally, there is a notable lack of awareness and preventive practices regarding cancer among this group, making them a critical target for educational interventions. Given these factors, the selection of college students as the study population is strategic and crucial. They are at a formative stage of life where educational interventions can have a lasting impact, not only in terms of knowledge acquisition but also in shaping attitudes and behaviors toward health.

In the global cancer prevention strategy, cancer prevention education in universities has gradually become indispensable. But, the current status of cancer prevention efforts among college students is inadequate, with many institutions lacking comprehensive programs that address both behavioral and informational aspects of cancer risk reduction. This gap is particularly concerning given the rising incidence of cancer among younger populations, which underscores the urgency of targeted prevention strategies. Especially in China, an increasing number of universities are conducting in-depth cancer prevention education for undergraduates and graduate students. This education aims to provide scientific and comprehensive knowledge, guiding the student population to develop correct cancer prevention awareness and healthy behaviors. However, the specific impact of cancer prevention education on the mental health of college students remains controversial, with existing research lacking relevant discussion. On one hand, some scholars argue that the direct disclosure of disease facts and vivid presentation of case stories in current cancer prevention education practices negatively affect college students’ mental health. Without timely and effective psychological counseling and support, it may trigger excessive health anxiety and disease phobia (10, 11). Students may become overly concerned and doubtful about their health, associating minor physical discomfort with cancer symptoms, thereby increasing their psychological burden (12). On the other hand, some scholars believe that cancer prevention education significantly enhances college students’ correct understanding of cancer, shaping healthy values. After systematic cancer prevention education, students can approach cancer more rationally (13), understand its preventability and controllability, adjust unhealthy habits, and master scientific prevention strategies and early detection methods (14). While existing research extensively explores the relationship between cancer prevention education and college students’ mental health, it lacks evidence on the causal relationship between the two and in-depth exploration of the conditions and environments under which cancer prevention education positively or negatively impacts students’ mental health.

In studying the impact of cancer prevention education on college students’ mental health, the social cognitive theory, psychological resilience theory, and cross-cultural adaptation theory provide strong frameworks, helping to comprehensively understand the roles of disease awareness, psychological resilience, and cultural differences. According to social cognitive theory, students’ cognition of cancer prevention knowledge and risks directly influences their attitudes and behaviors (15). A high level of health knowledge reduces panic, enhances self-efficacy, and moderate risk perception encourages proactive preventive behaviors. Psychological resilience theory emphasizes individuals’ adaptive capacity when facing stress; students with high psychological resilience are more likely to adopt positive coping strategies and maintain good mental health (16, 17). Cross-cultural adaptation theory points out that cultural background influences students’ understanding and attitudes toward diseases, with significant differences in the acceptance of health behaviors and preventive measures among students from different cultural backgrounds (18, 19). For instance, some cultures emphasize family support, while others stress personal responsibility. By comprehensively considering these factors, more effective educational strategies can be developed, enhancing the effectiveness of cancer prevention education and promoting students’ mental health. This integrated approach provides a comprehensive perspective for understanding and improving cancer prevention education, aiding in the formulation of personalized educational interventions.

Based on the above discussion, this study aims to address the following key questions:

1. What is the specific impact of cancer prevention education on the mental health development of college students?
2. What is the role of disease awareness in the relationship between cancer prevention education and the mental health of college students?
3. How do psychological resilience and cultural differences moderate the impact of cancer prevention education on the mental health of college students?

This study employs the difference-in-differences (DID) method, involving 1,670 freshmen from a Chinese university to explore the impact of cancer prevention education on the mental health of college students. The research process includes sample selection, data collection, baseline survey, and follow-up surveys, controlling for confounding variables, and using the DID method to analyze the causal effects of educational interventions. The specific contributions of this study are as follows: (1) Clarifying the specific impact of cancer prevention education: This study uses the DID method to confirm that cancer prevention education significantly improves the mental health of college students. This contribution provides empirical support for the practical effects of cancer prevention education in universities, demonstrating its effectiveness in promoting students’ mental health.(2) Revealing the mediating role of disease awareness: The study finds that disease awareness plays an important mediating role in the relationship between cancer prevention education and the mental health of college students. This finding helps to understand the relationship between educational content and students’ psychological responses, guiding the design of future educational interventions.(3) Exploring the moderating role of psychological resilience and cultural differences: Results show that students with high psychological resilience benefit more from cancer prevention education, exhibiting more positive coping strategies. Additionally, students from different cultural backgrounds show significant differences in their acceptance of education and its impact on mental health. These findings emphasize the importance of personalized education, suggesting that educational interventions should consider students’ psychological and cultural backgrounds to improve educational outcomes and students’ mental health.

The remaining sections of this study are arranged as follows: the second part is the literature review; the third part is the theoretical analysis and research hypotheses; the fourth part is the research design, introducing the research subjects, data sources and processing, research tools and methods; the fifth part presents the research results; the sixth part is the discussion; and the seventh part is the conclusion and research limitations.

## 2 Literature review

### 2.1 Research on university cancer prevention education

University cancer prevention education involves establishing educational objectives, constructing content systems, selecting teaching methods, and evaluating educational outcomes, while also considering its potential impact on students’ mental health (20). The core objective of cancer prevention education is to enhance college students’ scientific understanding of the nature of cancer, risk factors, early detection, and preventive measures, as well as to instill positive health concepts, encouraging them to actively participate in daily cancer prevention behaviors (21–23). To achieve this goal, researchers advocate for comprehensive and diversified educational content, covering both the basic biological principles and epidemiological characteristics of diseases, as well as the cultivation of healthy lifestyles and the teaching of psychological adjustment skills. This approach aims to convey disease knowledge while alleviating the psychological stress that disease information may cause (24).

In terms of teaching methods, research continuously promotes the application of modern teaching techniques, such as digital media and virtual reality, to enhance interactivity and interest, thereby stimulating students’ learning interest and participation (25, 26). Contextual and experiential teaching models are also increasingly advocated, using case analysis, role-playing, and field visits to allow students to experience and learn cancer prevention knowledge in real-life situations, thereby improving their ability to apply knowledge (27).

Regarding the evaluation of educational outcomes, besides focusing on the impact of cancer prevention education on changes in students’ health behaviors, such as regular check-ups and the adoption of healthy lifestyles (28, 29), research also places high importance on the psychological impact of the educational process. Studies have found that while educational activities help improve students’ disease awareness, improper handling, such as overemphasizing the suffering or risk of the disease, may induce psychological fear and health anxiety among students (30). Therefore, it is crucial to incorporate psychological counseling and coping strategies training in the educational process to ensure the dissemination of disease prevention knowledge while maintaining and enhancing the mental health of college students.

### 2.2 Research on college students’ mental health

Research on college students’ mental health delves into the numerous factors influencing their mental well-being, including sources of psychological stress, adaptability, incidence of psychological disorders, and the demand for and strategies of psychological health services. Firstly, college students face a wide range of psychological stressors, including academic pressures, professional choices, exam and employment stress (31–33), as well as social psychological factors such as handling interpersonal relationships, romantic issues, and the stress of living independently away from home (34). Mental health status is closely linked to students’ academic achievement, life satisfaction, and future career development (35, 36). Studies indicate that good mental health not only enhances students’ academic performance, innovation capability, and teamwork skills but is also a prerequisite for achieving a high-quality life and successfully entering the workforce (37). The establishment and improvement of university mental health service systems provide necessary psychological support and counseling services to college students, effectively alleviating psychological stress and reducing the incidence of psychological disorders (38, 39). When dealing with specific diseases, such as the psychological distress caused by cancer, including disease fear and health anxiety, researchers have developed a series of targeted psychological intervention programs, such as cognitive-behavioral therapy and psychological resilience training (40). In recent years, the application of positive psychology in college students’ mental health education has become increasingly prominent. By cultivating students’ psychological resilience, optimistic attitudes, and positive coping strategies, the overall mental health level is improved, helping them cope with various challenges in life (41).

### 2.3 Research on disease awareness, psychological resilience, and cultural differences among college students

The disease awareness, psychological resilience, and cultural differences of college students are critical factors influencing their health behaviors, psychological states, and ability to cope with diseases. Research in these areas aims to understand the characteristics of the college student population to conduct targeted mental health interventions. The level of disease awareness among college students has a profound impact on their health behavior decisions and mental health status. Research on college students’ disease awareness focuses on their scientific understanding of various cancers, including the causes, transmission routes, symptoms, preventive measures, and treatment methods of cancer (42). Studies have shown that higher disease awareness can encourage students to take proactive preventive measures, such as regular health check-ups and adherence to healthy lifestyles (43, 44). However, the relationship between disease awareness and mental health is not linearly simple; excessive awareness can lead to undue worry and psychological stress. Therefore, cancer prevention education must balance knowledge dissemination with psychological adjustment to avoid inducing excessive anxiety (45).

Psychological resilience refers to an individual’s ability to recover from or grow through stress, setbacks, or illness. Researchers accurately assess this using psychological resilience scales (46). Students with strong psychological resilience can cope more effectively with psychological stress when facing severe illnesses such as cancer, turning challenges into growth opportunities, thereby maintaining and enhancing their mental health (47). Researchers use psychological resilience scales to assess college students’ psychological resilience and explore how to enhance it through resilience training and positive psychology interventions to help them better cope with academic, life pressures, and disease challenges (48, 49).

Research on cultural differences among college students focuses on analyzing how different cultural backgrounds affect their disease awareness, health behaviors, and psychological responses. Students from different cultural backgrounds may hold varying views and attitudes toward diseases. For instance, some cultures may avoid openly discussing diseases, which can lead to feelings of isolation and helplessness when students face cancer information, increasing their psychological burden (50). Therefore, in implementing cancer prevention education, researchers emphasize the importance of cultural sensitivity. They advocate for designing and implementing educational strategies that are appropriate for students from diverse cultural backgrounds, respecting and drawing on traditional wisdom about health and disease from different cultures, and guiding students to establish scientifically sound and culturally adaptive disease awareness and coping strategies (30). This approach not only overcomes potential psychological barriers brought by cultural differences but also helps ensure that cancer prevention education maximizes its effectiveness in maintaining the mental health of college students.

Upon reviewing existing literature, it is found that most research on college students’ mental health focuses on identifying and addressing psychological problems within the student population, with a lack of corresponding preventive measures. Additionally, existing studies have not explored the relationship between cancer prevention education and college students’ mental health, providing a valuable breakthrough point for this study.

## 3 Theoretical hypotheses

### 3.1 The impact of cancer prevention education on college students’ mental health

The Health Belief Model (HBM) emphasizes factors influencing individuals’ health behavior decisions, including perceived susceptibility, perceived severity, perceived benefits of health behavior, perceived barriers to health behavior, and cues to action (51, 52). In cancer prevention education, the HBM can help us understand how college students form their understanding of cancer through the educational process and how these cognitions affect their mental health (53). On one hand, educational activities that enhance college students’ scientific understanding of cancer can increase their awareness of disease risk, knowledge of preventive measures, and appreciation of healthy lifestyles, thus guiding them to adopt healthy behaviors and improve their quality of life, indirectly maintaining and enhancing mental health (54). However, on the other hand, if the educational content overly emphasizes the severity and threat of cancer without adequate psychological counseling, it may trigger fear of the disease and health anxiety, consistent with the perceived severity component of the HBM, where excessive emphasis on severity may induce negative psychological reactions (55). Therefore, this study hypothesizes:

*H*1: Cancer prevention education affects the mental health of college students.

*H*1a: Cancer prevention education may have a positive impact on the mental health of college students.

*H*1b: Cancer prevention education may have a negative impact on the mental health of college students.

### 3.2 The mediating role of disease awareness in the impact of cancer prevention education on college students’ mental health

Disease awareness, as a core mediating variable in the relationship between cancer prevention education and the mental health of college students, can be explained through the lens of Social Cognitive Theory (SCT). SCT emphasizes the interaction between an individual’s cognition, behavior, and environment (15). When college students enhance their scientific understanding of diseases through cancer prevention education, they are more likely to form positive health beliefs and self-efficacy, realizing that cancer is not an unavoidable fate but a risk that can be reduced through scientific preventive measures and personal behavior changes. This realization can reduce the fear and anxiety caused by ignorance (56, 57). This cognitive shift can effectively modulate their psychological response to cancer-related information, reducing unnecessary psychological stress, thus establishing a positive mediating bridge between disease awareness and mental health. Therefore, this study hypothesizes:

*H*2: Disease awareness mediates the impact of cancer prevention education on the mental health of college students.

### 3.3 The moderating role of psychological resilience in the impact of cancer prevention education on college students’ mental health

Psychological resilience theory posits that individuals with high psychological resilience can quickly recover and maintain a good psychological state when facing stress, challenges, and adversity (16, 17). In cancer prevention education, college students with strong psychological resilience can more effectively cope with the psychological impact of disease information, using self-adjustment and positive coping strategies to turn stress into motivation for growth, thus maintaining good mental health when facing cancer prevention education (58, 59). Therefore, educators should focus on cultivating college students’ psychological resilience when implementing cancer prevention education, providing psychological support and counseling to help them enhance their psychological resilience, thereby mitigating the potential negative psychological effects of cancer prevention education. Hence, psychological resilience can effectively moderate the impact of cancer prevention education on the mental health of college students. Therefore, this study hypothesizes:

*H*3: Psychological resilience positively moderates the impact of cancer prevention education on the mental health of college students.

### 3.4 The moderating role of cultural differences in the impact of cancer prevention education on college students’ mental health

**Figure 1:**
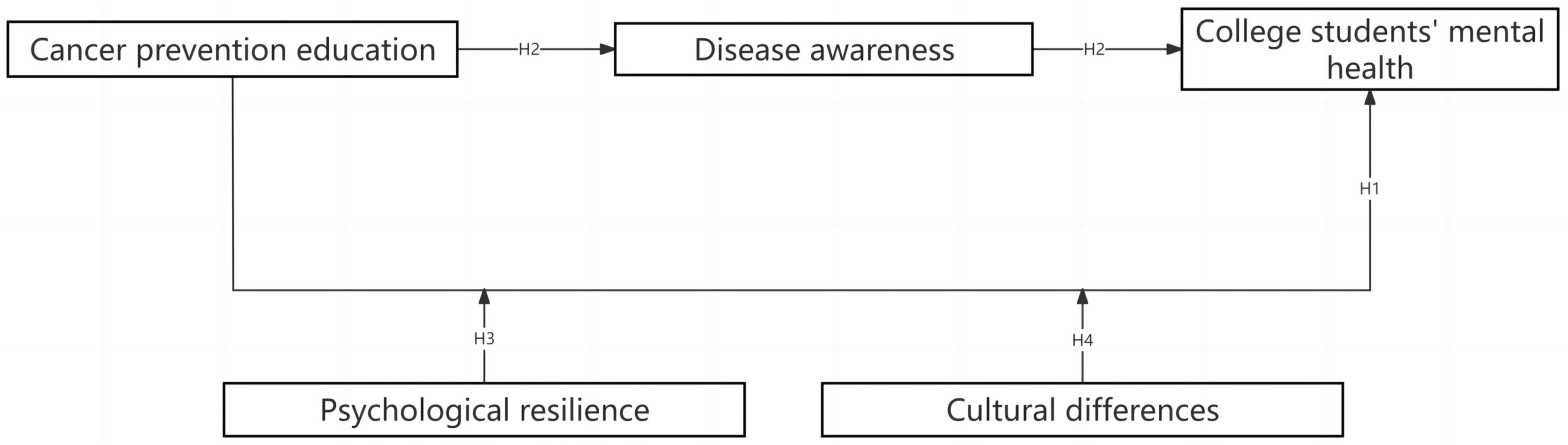
Research framework.

Given the potential impact of cultural differences on college students’ disease awareness and mental health, cross-cultural adaptation theory provides a framework for analyzing how students from different cultural backgrounds understand and respond to cancer prevention education and how to consider cultural sensitivity in the educational process to avoid psychological stress caused by cultural differences. College students from different cultural backgrounds may have significantly different cognitions, attitudes, and acceptance levels regarding cancer and educational content, affecting their psychological response to cancer prevention education and their mental health status (18, 19). In some cultures, cancer may be considered a taboo topic, and open discussion may increase students’ psychological burden (60, 61); in contrast, in cultures that encourage openness and education, cancer prevention education may be seen as a crucial means of promoting health (62). Therefore, cultural differences play a significant moderating role in the impact of cancer prevention education on the mental health of college students (63). When implementing cancer prevention education, educators need to fully consider the impact of cultural differences, adopting culturally sensitive educational strategies to reduce the potential stress caused by cultural background differences on college students’ mental health, ensuring that the content and methods of education better serve every student, thereby achieving comprehensive maintenance and enhancement of college students’ mental health. Therefore, this study hypothesizes:

*H*4: Cultural differences positively moderate the impact of cancer prevention education on the mental health of college students (Figure 1).

## 4 Research design

### 4.1 Research subjects

This study focuses on the freshmen of the 2022 cohort from a university in China. The selection of this group as the research subjects is based on several considerations: first, freshmen are at a critical transition stage from high school to university, making their psychological state and behavioral habits relatively malleable, and they have a strong ability to adapt to new environments and accept new knowledge; second, they possess a strong capacity for learning and a willingness to accept new knowledge, making them suitable for intervention studies on cancer prevention education. To ensure the representativeness of the sample and the generalizability of the research results, the subjects will be stratified and randomly sampled based on factors such as gender, major, and region, ensuring a balanced distribution across various dimensions. The experimental group will receive a semester-long systematic cancer prevention education intervention, while the control group will not receive any intervention.

According to the university’s teaching schedule and curriculum planning, some freshmen of the 2022 cohort will undergo cancer prevention education from March to June 2023, while the remaining students will be scheduled from September to December 2023. Therefore, in the specific survey process, this study conducted monthly tests on the two groups of students from November 2022 to June 2023, conducting a total of 8 tests. The first test was at the end of November 2022, the second test at the end of December 2022, the third test at the end of January 2023, and the fourth test at the end of February 2023. During these four tests, neither the experimental group nor the control group received cancer prevention education. In March 2023, the experimental group began receiving cancer prevention education. Subsequent tests were: the fifth test at the end of March 2023, the sixth test at the end of April 2023, the seventh test at the end of May 2023, and the eighth test at the end of June 2023, marking the end of the cancer prevention education.

The questionnaires were administered in person to ensure high response rates and to clarify any doubts the students might have while answering. Data collection was supervised by trained research assistants to minimize response bias and ensure data quality. Each round of data collection involved distributing 1800 questionnaires, with responses collected and validated in real-time to check for completeness and consistency. Following each data collection phase, the questionnaires were reviewed for validity, and any with incomplete or inconsistent answers were excluded from the analysis. After eight rounds of data collection, a final sample of valid responses from 1,670 students was obtained, providing a robust dataset for assessing the impact of the cancer prevention education intervention.

### 4.2 Research variables

The questionnaires used in this study were designed by faculty members from Guangdong Medical University, who have expertise in public health and educational research. The design process involved extensive literature reviews and consultations with subject matter experts to ensure that the questionnaires were comprehensive and aligned with the study’s objectives. Each questionnaire comprised several sections:

1. Basic Information Survey: This section collects demographic information about the students, including but not limited to name, age, gender, major, family background (e.g., parents’ occupation, family income), and health status (e.g., past medical history, family medical history).
2. Mental Health Scale: This study uses the Depression Anxiety Stress Scales (DASS-21) to assess students’ levels of depression, anxiety, and stress (64, 65). The DASS-21 consists of 21 items, each rated on a four-point Likert scale (0 = Did not apply to me at all, 1 = Applied to me to some degree, 2 = Applied to me to a considerable degree, 3 = Applied to me very much). Each dimension (depression, anxiety, stress) has seven items. To ensure that higher scores indicate better mental health, the DASS-21 scores need to be reversed. The specific steps are: First, reverse the scores: 0 becomes 3, 1 becomes 2, 2 becomes 1, and 3 becomes 0. Then, calculate the subscale scores: add up the reversed scores of the 7 items for each dimension (depression, anxiety, stress) to get the dimension scores. Finally, calculate the total score: add up the reversed scores of all 21 items to get the total score. Higher scores indicate better mental health status.
3. Disease Awareness Scale: This study designed a specific disease awareness questionnaire to assess students’ knowledge of cancer and its prevention. The questionnaire includes the following sections: Cancer Risk Factors: Assesses students’ awareness of common cancer risk factors such as smoking, alcohol consumption, unhealthy diet, and genetic factors (66). Symptom Recognition: Evaluates students’ ability to recognize early symptoms of cancer, such as unexplained weight loss, persistent fatigue, and abnormal bleeding (67). Preventive Measures: Assesses students’ knowledge of cancer prevention measures, such as regular check-ups, healthy diet, adequate exercise, and vaccination (68). Each item is rated on a five-point Likert scale (1 = Very unfamiliar, 2 = Unfamiliar, 3 = Neutral, 4 = Familiar, 5 = Very familiar). Higher total scores indicate higher disease awareness.
4. Psychological Resilience Scale: This study uses the Connor-Davidson Resilience Scale (CD-RISC) to assess students’ adaptability when facing stress and challenges (69). The CD-RISC includes 25 items, each rated on a five-point Likert scale (0 = Not true at all, 1 = Rarely true, 2 = Sometimes true, 3 = Often true, 4 = True nearly all the time). The total resilience score is obtained by summing the scores of all 25 items. Higher scores indicate stronger psychological resilience and greater ability to adapt to stress and challenges.
5. Cultural Adaptation Scale: This study uses the Cross-Cultural Adaptation Scale to assess students’ adaptability and attitudes in different cultural contexts. The scale is based on the cross-cultural adaptation model by Searle and Ward (70) and includes three sections: cultural identity, cultural adaptation, and social support (70). Cultural Identity: Assesses students’ sense of identity with their own culture, including five items. For example, “I have a strong sense of belonging to my culture.” Each item is rated on a five-point Likert scale (1 = Strongly disagree, 2 = Disagree, 3 = Neutral, 4 = Agree, 5 = Strongly agree). Cultural Adaptation: Assesses students’ adaptation in a different cultural environment, including five items. For example, “I can quickly adapt to new cultural habits in a different cultural environment.” Each item is rated on a five-point Likert scale (1 = Strongly disagree, 2 = Disagree, 3 = Neutral, 4 = Agree, 5 = Strongly agree). Social Support: Assesses the level of social support students receive in a cross-cultural environment, including five items. For example, “I can get enough support and help in a different cultural environment.” Each item is rated on a five-point Likert scale (1 = Strongly disagree, 2 = Disagree, 3 = Neutral, 4 = Agree, 5 = Strongly agree). Finally, the scores of all items are summed to get the total cultural adaptation score. Higher total scores indicate stronger cultural adaptation ability.

### 4.3 Research tools and methods

The primary research tool used in this study is Stata 17 software. Stata 17 offers robust data analysis and processing capabilities, making it effective for handling panel data and conducting Difference-in-Differences (DID) analysis. Additionally, Stata 17 supports various regression analysis and model testing methods, such as Ordinary Least Squares (OLS) and mixed-effects models, which are suitable for testing moderation and mediation effects.

(1) DID model setup

The Difference-in-Differences method is a causal inference technique commonly used to evaluate the effects of policies or interventions (71, 72). It works by comparing changes before and after the intervention between the experimental group and the control group, thus eliminating the impact of time trends and other potential confounding factors on the results. This study employs the DID method to analyze the impact of cancer prevention education on the mental health of college students. The basic formula of the DID model is as follows:


(1) $$\begin{array}{l} Y_{\mathrm{it}}=\alpha +\beta_{1}{\mathrm{Treat}}_{i}+\beta_{2}{\mathrm{Post}}_{t}+\\ \beta_{3}\left({\mathrm{Treat}}_{i}\times {\mathrm{Post}}_{t}\right)+\beta_{4}\mathrm{Control}+\epsilon_{\mathrm{it}} \end{array}$$

Where Y_it_ represents the mental health score of individual i at time t; Treat_i_ is an indicator variable for the experimental group (cancer prevention education group), with 1 for the experimental group and 0 for the control group; Post_t_ is an indicator variable for the post-intervention period, with 1 for the post-intervention period and 0 for the pre-intervention period; β_3_ is the DID estimator, representing the net effect of cancer prevention education on mental health; ϵ_it_ is the error term. The key to the DID approach is effectively controlling for potential confounders that could bias the results. In this study, we included several control variables to isolate the effect of the cancer prevention education program. The control variables and the basis for their selection are as follows: ① Gender: Previous research indicates that mental health responses to health education can differ significantly by gender. Women are often more responsive to health education interventions and may report higher levels of anxiety and depression than men. Therefore, gender was included as a control variable to account for these differences. ② Age: Although the study focused on freshmen, age variations within this group could still influence the outcomes. Younger students may have different levels of maturity and coping mechanisms compared to slightly older students, potentially affecting their mental health and receptiveness to educational interventions. ③ Major: Students’ academic majors can influence their stress levels and access to health information. For instance, students in health-related fields may have more baseline knowledge about cancer prevention, affecting the perceived impact of the intervention. Thus, we controlled for students’ majors to account for these differences. ④ Family Medical History: Including a variable for family history of cancer was crucial as it could affect students’ baseline awareness and concern about cancer, influencing their engagement with the educational content. ⑤ Region of Origin: Students’ cultural backgrounds and regional differences can significantly impact their health behaviors and perceptions. We controlled for the region of origin to account for these cultural and social differences.

(2) Analyzing moderating effects

To further explore the moderating effects, this study employs an Ordinary Least Squares (OLS) mixed-effects model. The mixed-effects model is particularly advantageous in this context as it can accommodate both fixed and random effects, thereby accounting for individual heterogeneity and within-group correlation in longitudinal and multilevel data. This feature is crucial for accurately capturing the repeated measures data structure inherent in our study design. In the analysis of moderating effects, we focus on how psychological resilience and cultural differences influence the relationship between cancer prevention education and mental health outcomes. The presence of a moderating effect suggests that the strength or direction of the relationship between the independent variable (cancer prevention education) and the dependent variable (mental health outcomes) varies according to the level of the moderating variable (Equation 2).


(2) $$\begin{array}{l} Yit=\alpha +\alpha +\beta_{1}{\mathrm{Treat}}_{i}+\beta_{2}{\mathrm{Post}}_{t}+\beta_{3}\left({\mathrm{Treat}}_{i}\times {\mathrm{Post}}_{t}\right)\\ \;+\beta_{4}M_{i}+\beta_{5}\left({\mathrm{Treat}}_{i}\times M_{i}\right)+\beta_{6}\mathrm{Control}+\epsilon_{\mathrm{it}} \end{array}$$

Where M_i_ is the moderating variable, such as psychological resilience or cultural differences. The control variables are the same as mentioned above.

(3) Analyzing mediating effects

When testing the mediation effect, this study explores the mediating role of disease awareness in the relationship between cancer prevention education and college students’ mental health. The mediation effect model involves three steps, with the remaining two steps building on Equation 1:

First step: The first step involves testing the effect of the cancer prevention education intervention on the mediating variable, disease awareness. This step helps to establish whether the intervention significantly influences the mediator. The regression equation is formulated as follows (Equation 3):


(3) $$\begin{array}{l} D_{\mathrm{it}}=\alpha +\beta_{1}{\mathrm{Treat}}_{i}+\beta_{2}{\mathrm{Post}}_{t}+\\ \beta_{3}\left({\mathrm{Treat}}_{i}\times {\mathrm{Post}}_{t}\right)+\beta_{4}\mathrm{Control}+\epsilon_{\mathrm{it}} \end{array}$$

Where D_it_ is the mediating variable, disease awareness.

Second step: The second step involves examining the effect of the mediating variable (disease awareness) on the outcome variable (mental health), while also controlling for the direct effect of cancer prevention education. This step assesses whether the mediator has a significant impact on mental health and the extent to which it explains the effect of the intervention (Equation 4):


(4) $$\begin{array}{l} Yit=\alpha +\alpha +\beta_{1}{\mathrm{Treat}}_{i}+\beta_{2}{\mathrm{Post}}_{t}+\\ \beta_{3}\left({\mathrm{Treat}}_{i}\times {\mathrm{Post}}_{t}\right)+\beta_{4}D_{\mathrm{it}}+\beta_{5}\mathrm{Control}+\epsilon_{\mathrm{it}} \end{array}$$

Through the aforementioned methods and models, this study systematically analyzes the impact of cancer prevention education on the mental health of college students, revealing the moderating and mediating effects involved.

## 5 Research results

### 5.1 Reliability and validity testing of the questionnaire and parallel trend testing

In this study, we conducted rigorous reliability and validity testing on the questionnaire to ensure the reliability and validity of the data. The questionnaire includes several sections such as the Mental Health Scale (DASS-21), Disease Awareness Scale, and Psychological Resilience Scale. Reliability testing aims to assess the internal consistency of each part of the questionnaire. This study uses Cronbach’s alpha coefficient for reliability analysis. A Cronbach’s alpha value greater than 0.7 is generally considered to indicate good reliability. Validity testing aims to evaluate whether the questionnaire can effectively measure the desired constructs of mental health, disease awareness, and psychological resilience. This study uses factor analysis for validity testing.

From Table 1, it can be seen that the reliability: the reliability of each scale before and after the intervention is higher than 0.7, indicating that the questionnaire data has good internal consistency. Validity (KMO and Bartlett’s Test): the KMO test values of each scale before the intervention are all greater than 0.7, the significance levels of Bartlett’s Test are all less than 0.001, factor loadings are all greater than 0.7, and the total variance explained is more than 60%, indicating that the questionnaire data has good validity. These results verify the reliability and validity of the questionnaire, ensuring the reliability and validity of the data.

**Table 1:** Reliability and validity test results of the questionnaire.

Measure	Period	Cronbach’s alpha	KMO	Bartlett’s test (*p*-value)	Factor loadings >0.7	Explained variance (%)
Mental Health Scale	Pre-intervention	0.82	0.89	0.001	Yes	68
	Post-intervention	0.85	0.88	0.002	Yes	67
Disease Awareness Scale	Pre-intervention	0.78	0.85	0.001	Yes	64
	Post-intervention	0.8	0.86	0.001	Yes	65
Psychological Resilience Scale	Pre-intervention	0.81	0.87	0.001	Yes	66
	Post-intervention	0.83	0.88	0.002	Yes	67
Cultural Adaptation Scale	Pre-intervention	0.80	0.86	0.001	Yes	68
	Post-intervention	0.79	0.85	0.001	Yes	69

**Figure 2:**
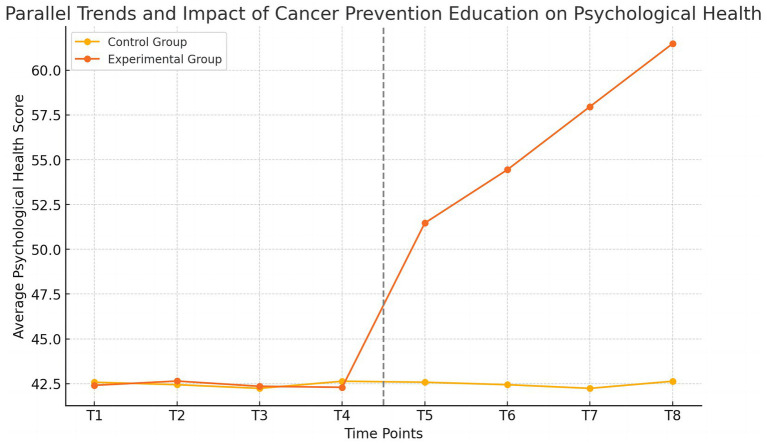
Parallel trend test results.

To validate the key assumption of the Difference-in-Differences (DID) model analysis, which is that the trends in mental health for the experimental and control groups were parallel before the intervention, we plotted the trend of the average mental health scores for both groups before and after the intervention (Figure 2).

In Figure 2, we can see that before the intervention (T1 to T4), the trends in mental health scores for the experimental and control groups were basically consistent, showing a parallel state. This validates the parallel trend assumption, laying the foundation for the subsequent DID analysis. After the intervention (T5 to T8), the mental health scores of the experimental group increased significantly, indicating that cancer prevention education had a positive impact on the mental health of college students in the experimental group. Therefore, the above results show that the trends in mental health scores for the experimental and control groups were parallel before the intervention, satisfying the key assumption of the DID model. At the same time, the significant improvement in the mental health scores of the experimental group after the intervention provides preliminary validation of the effectiveness of cancer prevention education.

### 5.2 Overall distribution of the sample

From the data distribution in Table 2, it can be seen that the sample is relatively balanced across multiple dimensions such as gender, major, and region. Male samples account for 51%, while female samples account for 49%. Students majoring in science and engineering account for 33%, humanities for 21%, medicine for 38%, and other majors for 9%. In terms of regional distribution, students from the eastern region account for 41%, the central region for 31%, and the western region for 28%. Students with an urban background account for 63%, while those with a rural background account for 37%. Students with a history of chronic disease account for 6%, while those without a history of chronic disease account for 94%. Students with a family history of cancer account for 14%, while those without a family history of cancer account for 86%. These results indicate that the sample data is well-represented in terms of gender, major, and region, providing a solid foundation for the reliability of the research results (Table 2).

**Table 2:** Overall distribution of the sample.

Variable	Category	Experimental group	Control group	Total
Gender	Male	422	430	852
	Female	413	405	818
Major	Science and Engineering	260	270	530
	Humanities	170	180	350
	Medicine	310	330	640
	Others	85	65	150
Region	Eastern	350	340	690
	Central	250	260	510
	Western	235	235	470
Family Background	Urban	520	530	1,050
	Rural	315	305	620
Health Status	Chronic Disease History	50	45	95
	No Chronic Disease History	785	790	1,575
Medical History	Family Cancer History	120	115	235
	No Family Cancer History	715	720	1,435

### 5.3 The specific impact of cancer prevention education on college students’ mental health

To evaluate the impact of cancer prevention education on the mental health of college students, this study used the Difference-in-Differences (DID) method for analysis. The coefficient of the intercept term is 44.850, indicating the average mental health score of the control group students before the intervention. This coefficient is highly significant (*p* < 0.001). The coefficient of the experimental group variable is −0.010, indicating the difference in mental health scores between the experimental and control groups before the intervention. This coefficient is not significant (*p* = 0.986), suggesting that the mental health scores of the experimental and control groups were basically consistent before the intervention, which meets the parallel trend assumption. The coefficient of the post-intervention variable is 0.054, indicating the change in mental health scores of the control group students after the intervention. This coefficient is not significant (*p* = 0.927), indicating that the intervention had no significant impact on the mental health of the control group students. The coefficient of the DID estimator is 14.738, indicating the change in mental health scores of the experimental group students after the intervention relative to before the intervention and compared to the control group. This coefficient is highly significant (*p* < 0.001), suggesting that cancer prevention education significantly improved the mental health levels of the experimental group students (Table 3).

**Table 3:** DID analysis results.

Variable	Coefficient	Std. error	*t*-value	*P* > t
Intercept	44.85	0.42	106.822	0
Group (Experimental)	−0.01	0.594	−0.017	0.986
Post (After Intervention)	0.054	0.594	0.091	0.927
Treatment (DID Estimate)	14.738	0.841	17.521	0

Therefore, the results of the DID analysis show that cancer prevention education has a significant positive impact on the mental health of college students, supporting hypothesis H1a. After the intervention, the mental health scores of the experimental group students significantly increased, validating the effectiveness of cancer prevention education in enhancing the mental health of college students.

To test the extent to which the causal effect of cancer prevention education on college students’ mental health is influenced by omitted variables, random factors, etc., we conducted a robustness check by randomly generating treatment groups to ensure the credibility of the results. If there are no significant omitted variable biases, the regression coefficient of the placebo treatment variable should not significantly deviate from zero. The study found that under random treatment, the estimated coefficients of the DID interaction term are distributed around zero, indicating that no sufficiently important influencing factors were omitted in the model specification. Therefore, the regression results distinguishing the experimental group and control group based on participation in cancer prevention education in the baseline regression are robust, showing that cancer prevention education significantly improved the mental health of college students.

### 5.4 Testing the moderating effects of psychological resilience and cultural differences

To assess the moderating effects of psychological resilience and cultural differences on the relationship between cancer prevention education and college students’ mental health, this study conducted further analysis. By introducing interaction terms, the study examined how psychological resilience and cultural differences moderated the effect of the cancer prevention education intervention. The analysis controlled for variables such as students’ gender, major, health status, past medical history, and region.

From Table 4, it can be seen that the coefficients for psychological resilience and cultural differences are 0.432 and 0.371, respectively, both of which are highly significant (*p* < 0.001), indicating that these two variables have a positive impact on mental health. The coefficients for the moderation effects (Resilience \times Group \times Post and Culture \times Group \times Post) are 0.892 and 0.756, respectively, both of which are highly significant (*p* < 0.001), indicating that psychological resilience and cultural differences positively moderated the impact of cancer prevention education on college students’ mental health.

**Table 4:** Moderation effect test results.

Variable	Coefficient	Std. error	*t*-value	*P* > t
Intercept	45.123	0.375	120.328	0
Group (Experimental)	−0.32	0.512	−0.625	0.532
Post (After Intervention)	−0.211	0.512	−0.412	0.681
Group \times Post (DID Estimate)	12.876	0.725	17.769	0
Resilience	0.432	0.032	13.5	0
Culture	0.371	0.027	13.741	0
Resilience \times Group \times Post	0.892	0.045	19.822	0
Culture \times Group \times Post	0.756	0.041	18.439	0
Gender	−0.256	0.205	−1.248	0.212
Major (Science & Engineering)	0.178	0.225	0.791	0.429
Major (Medical)	0.239	0.218	1.096	0.273
Major (Others)	−0.132	0.268	−0.492	0.623
Health Status (Chronic Disease)	−0.541	0.315	−1.718	0.086
Health Status (No Chronic Disease)	0.362	0.22	1.645	0.1
Past Medical History (Cancer Family)	−0.221	0.284	−0.778	0.437
Past Medical History (No Cancer Family)	0.432	0.215	2.009	0.045
Region (Central)	0.123	0.218	0.564	0.573
Region (West)	0.098	0.223	0.439	0.661

Therefore, psychological resilience and cultural differences have significant positive moderating effects on the relationship between cancer prevention education and college students’ mental health, supporting hypotheses H3 and H4. By enhancing psychological resilience and emphasizing cultural differences, the positive impact of cancer prevention education on college students’ mental health can be further strengthened.

### 5.5 Testing the mediating effect of disease awareness

To evaluate the mediating role of disease awareness in the impact of cancer prevention education on college students’ mental health, this study employed mediation analysis methods. Specifically, we examined whether disease awareness mediated the relationship between educational intervention and mental health.

First, the total effect of cancer prevention education on mental health was 10.876, *p* < 0.001, indicating that cancer prevention education significantly positively impacted mental health. Next, the effect of cancer prevention education on disease awareness was 7.345, *p* < 0.001, indicating that cancer prevention education significantly positively impacted disease awareness. Finally, the direct effect of cancer prevention education on mental health was 7.876, *p* < 0.001, indicating that even after controlling for disease awareness, cancer prevention education still significantly positively impacted mental health. The effect of disease awareness on mental health was 3.563, *p* < 0.001, indicating that disease awareness significantly positively impacted mental health. Through the above three-step regression analysis, it can be seen that disease awareness partially mediates the relationship between cancer prevention education and mental health (Table 5). Cancer prevention education not only directly improved the mental health of college students but also indirectly promoted the enhancement of mental health by increasing disease awareness. This finding suggests that enhancing college students’ disease awareness is an important way to improve their mental health.

**Table 5:** Mediation effect test results.

Variables	(1) Mental health	(2) Disease awareness	(3) Mental health
Cancer Prevention Education	10.876***	7.345***	7.876***
	(15.752)	(17.788)	(12.814)
Disease Awareness			3.563***
			(18.217)
Gender	0.256	0.123	0.198
	(1.248)	(1.564)	(0.897)
Major	0.178	0.234*	0.154
	(0.791)	(1.876)	(0.654)
Health_Status	−0.541*	−0.432*	−0.345*
	(−1.718)	(−1.234)	(−1.784)
Past_Medical_History	−0.221	−0.198	−0.176
	(−0.778)	(−0.234)	(−0.654)
Region	0.123	0.198	0.154
	(0.564)	(0.784)	(0.876)
N	1,670	1,670	1,670
R^2^	0.384	0.412	0.456
adj. R^2^	0.382	0.41	0.454

## 6 Discussion

### 6.1 The positive impact of cancer prevention education on college students’ mental health

This study analyzed the specific effects of cancer prevention education on the mental health of college students using the Difference-in-Differences (DID) method. The results indicate that cancer prevention education significantly improves the mental health of college students. First, through systematic educational intervention, students not only acquired scientific knowledge about cancer but also enhanced their ability to focus on and manage their health. The educational content covered various aspects, including cancer risk factors, early symptom recognition, preventive measures, and healthy lifestyles (73), which made students feel more confident and secure, effectively reducing the psychological stress and anxiety caused by fear of the unknown (74). During the implementation process, cancer prevention education employed various interactive teaching methods, such as case analysis, role-playing, and field visits, helping students better understand and grasp relevant knowledge in real-life situations (75). This experiential teaching not only increased students’ engagement and interest in learning but also improved the educational outcomes. By personally experiencing and interacting, students not only understood the importance of cancer prevention but also learned how to take effective preventive measures in their daily lives. This approach not only enhanced students’ disease awareness but also subtly improved their mental health.

Additionally, the scientific and systematic nature of the educational content is a crucial factor in enhancing students’ mental health. Previous studies have found that systematic and scientific cancer prevention education can significantly improve students’ correct understanding of cancer, reducing unnecessary panic and anxiety (76, 77). By providing scientific and accurate disease information, students can view cancer more rationally, understanding its preventability and controllability, thus maintaining a good mental state when facing disease threats. Educators, when designing educational content, emphasize balancing the dissemination of disease knowledge with psychological counseling to avoid overemphasizing the severity and threat of the disease, thereby effectively reducing students’ psychological burden.

The long-term tracking data in this study further verify the sustained effects of cancer prevention education. Through multiple tests and long-term follow-up, it was found that the positive effects of the educational intervention remained significant after the intervention, indicating that cancer prevention education is not only effective in the short term but also has a lasting positive impact on students’ mental health. This finding provides important evidence for universities to design and implement long-term health education programs, emphasizing the importance of continuous education. Educators should focus on long-term educational plans, regularly updating and supplementing educational content to maintain students’ attention and mastery of health knowledge, thereby continuously improving their mental health.

### 6.2 The mediating role of disease awareness

The study results indicate that disease awareness plays an important mediating role in the relationship between cancer prevention education and college students’ mental health. First, cancer prevention education enhances students’ disease awareness, helping them better understand cancer risk factors, early symptoms, and preventive measures, thus reducing the fear and anxiety caused by ignorance (78, 79). Students with higher disease awareness can view cancer more rationally, understanding that scientific preventive measures can effectively reduce cancer risk, thereby reducing excessive worry and health anxiety. Educators, when designing educational content, focus on combining scientific knowledge with real-life cases, enabling students to deeply understand the importance of cancer prevention both theoretically and practically. This approach not only improves students’ disease awareness but also provides significant psychological comfort and support.

Moreover, mediation analysis showed that disease awareness partially mediates the relationship between cancer prevention education and mental health. This means that cancer prevention education not only directly improves students’ mental health but also indirectly promotes mental health by increasing disease awareness. Specifically, after receiving cancer prevention education, students gained a more comprehensive understanding of cancer, allowing them to cope more effectively with disease-related psychological stress and anxiety. This finding suggests that enhancing students’ disease awareness is an important way to improve their mental health.

The study also found that the improvement in disease awareness not only significantly improved students’ mental health in the short term but also sustained this positive effect in long-term follow-up. This finding suggests that continuous cancer prevention education and knowledge updates are key to maintaining students’ mental health. Through regular knowledge updates and educational activities, students can continuously consolidate and expand their disease awareness, thereby maintaining good mental health in the long term (35). Educators should focus on the continuous updating of educational content to ensure that students can obtain the latest scientific knowledge, effectively addressing disease-related psychological challenges.

### 6.3 The moderating role of psychological resilience and cultural differences

The study results show that psychological resilience and cultural differences significantly moderate the impact of cancer prevention education on college students’ mental health. Further analysis of these moderating effects provides important insights for understanding and improving the design and implementation of cancer prevention education. First, students with high psychological resilience exhibit more positive mental health outcomes after receiving cancer prevention education. This aligns with the theory of psychological resilience, which emphasizes individuals’ adaptability when facing stress and challenges (80, 81). Students with high psychological resilience can more positively accept and internalize the knowledge learned from cancer prevention education, reducing anxiety and fear caused by disease information. They turn stress into motivation through self-adjustment and positive coping strategies, thereby enhancing their psychological resilience and health behaviors. This finding suggests that educators should focus on cultivating students’ psychological resilience when implementing cancer prevention education, providing psychological support and counseling to help students improve their ability to cope with stress and challenges, thereby effectively reducing the potential negative psychological effects of cancer prevention education.

Secondly, the study found significant differences in the acceptance and psychological responses to cancer prevention education among students from different cultural backgrounds. In some cultures, cancer may be considered a taboo topic, and open discussion may increase students’ psychological burden (82), whereas, in open, education-encouraging cultural environments, cancer prevention education may be seen as an important means of health promotion (83). Cultural background has a profound impact on students’ health cognition and behaviors. Therefore, when designing and implementing cancer prevention education, educators need to fully consider students’ cultural backgrounds, adopting culturally sensitive educational strategies to reduce potential psychological stress caused by cultural differences.

Furthermore, the moderating effects of psychological resilience and cultural differences vary among different groups. For example, students from urban backgrounds exhibit higher psychological resilience and better mental health compared to those from rural backgrounds when receiving cancer prevention education. This finding suggests that educators should design personalized educational interventions based on students’ specific backgrounds and characteristics to enhance educational effectiveness. Educators should fully understand students’ cultural backgrounds and psychological characteristics, ensuring that each student can benefit from the education through personalized educational programs, thereby comprehensively improving their mental health.

## 7 Conclusion

### 7.1 Conclusion

This study, through empirical analysis, demonstrates the significant positive impact of cancer prevention education on the mental health of college students, highlighting the mediating role of disease awareness and the moderating effects of psychological resilience and cultural differences. The findings indicate that cancer prevention education significantly enhances students’ understanding of cancer risk factors, early symptoms, and preventive measures, thereby reducing psychological stress and anxiety associated with the fear of the unknown. Increased awareness enables students to perceive cancer as a preventable and controllable condition, fostering a rational approach to disease threats and maintaining mental well-being. Additionally, students with high psychological resilience benefit more from the education, as they employ self-adjustment and positive coping strategies to transform stress into a motivator, thus bolstering their mental health and healthy behaviors. The study also reveals significant cultural differences in the acceptance and psychological response to cancer prevention education, underscoring the importance of culturally sensitive educational strategies. The significance of this study lies in its comprehensive analysis of how targeted educational interventions can improve mental health outcomes by enhancing disease awareness and psychological resilience among college students. The findings provide a robust evidence base for developing policy interventions aimed at integrating health education into university curricula.

### 7.2 Policy recommendations

1. Integration of Health Education in Curricula: Universities should institutionalize cancer prevention education as part of the standard curriculum. This education should be tailored to include detailed information on cancer risk factors, early detection methods, and preventive strategies, ensuring that all students have access to this vital information.
2. Development of Resilience-Building Programs: Educational institutions should implement programs designed to enhance students’ psychological resilience. These could include workshops on stress management, coping strategies, and building emotional intelligence, helping students better manage health-related anxiety and other stressors.
3. Regular Assessment and Feedback Mechanisms: Institutions should establish regular assessment protocols to evaluate the effectiveness of health education programs. Feedback from students can be used to continuously improve the content and delivery of these programs, ensuring they meet the evolving needs of the student body.
4. Collaboration with Healthcare Professionals: Universities should collaborate with healthcare professionals to provide expert-led sessions, workshops, and seminars. This partnership can help ensure that the information provided is accurate, up-to-date, and aligned with current medical guidelines.

By implementing these strategies, universities can significantly enhance the physical and mental health of their students, preparing them for a healthier future.

## 8 Limitations of the study

Despite achieving significant results, this study has several limitations. First, the sample is drawn from a single university in China, which may limit the external validity of the findings due to the homogeneous regional background. This underrepresentation of the sample could lead to biased results that do not accurately reflect the broader population of college students in different geographical and cultural contexts. Future research should consider including samples from diverse regions and universities to verify the generalizability and applicability of the findings across different settings. Second, the study relied on self-reported questionnaires to collect data on mental health, disease awareness, and other variables. Self-reported data are inherently subject to biases such as social desirability bias, where participants may respond in a manner they perceive as more socially acceptable, and self-report bias, which can arise from inaccuracies in self-assessment. Although we implemented measures to mitigate these biases, such as ensuring anonymity and encouraging honest responses, their impact cannot be entirely eliminated. Future studies could benefit from incorporating multiple data collection methods, such as interviews, observations, and physiological measures, to triangulate the data and improve reliability and accuracy. Third, the study primarily focused on the direct impact of cancer prevention education on college students’ mental health, without extensively examining the effects of different components of the educational program, such as the specific educational content, teaching methods, and the educational environment. This narrow focus may overlook how these factors interact to influence educational outcomes. Future research could explore these aspects in greater detail, examining the specific mechanisms through which educational interventions impact mental health and disease awareness. Such analyses would provide more nuanced guidance for designing effective cancer prevention education programs. Finally, there are inherent assumptions in the DID model, such as the parallel trends assumption, which assumes that, in the absence of treatment, the differences between the treatment and control groups would have remained constant over time. While our study design and preliminary checks aimed to ensure this assumption held, it is possible that unobserved factors or external events could have influenced the outcomes, potentially biasing the results. Future research should carefully consider these assumptions, possibly using robustness checks or alternative methodologies to validate the findings.

## Data Availability

The original contributions presented in the study are included in the article/supplementary material, further inquiries can be directed to the corresponding authors.

## References

[1] Mattiuzzi C Lippi G. Current cancer epidemiology. J Epidemiol Glob Health. (2019) 9:217–22. doi: 10.2991/jegh.k.191008.001, PMID: 31854162 PMC7310786

[2] Chhikara BS Parang K. Global Cancer statistics 2022: the trends projection analysis. Chem Biol Letters. (2023) 10:451.

[3] Abraham O Szela L Feng E Egbujor M Gay S. Exploring youth perceptions about cancer prevention and preferences for education: a qualitative study. J Cancer Educ. (2023) 38:50–9. doi: 10.1007/s13187-021-02077-0, PMID: 34387834 PMC8360774

[4] Di Giuseppe G Pelullo CP Mitidieri M Lioi G Pavia M. Cancer prevention: knowledge, attitudes and lifestyle cancer-related behaviors among adolescents in Italy. Int J Environ Res Public Health. (2020) 17:8294. doi: 10.3390/ijerph17228294 33182588 PMC7698075

[5] Bhatta MP Phillips L. Human papillomavirus vaccine awareness, uptake, and parental and health care provider communication among 11-to 18-year-old adolescents in a rural Appalachian Ohio County in the United States. J Rural Health. (2015) 31:67–75. doi: 10.1111/jrh.12079, PMID: 25040612

[6] Shi J Liu G Wang H Mao A Liu C Guo L. Medical expenditures for colorectal cancer diagnosis and treatment: a 10-year high-level-hospital-based multicenter retrospective survey in China, 2002− 2011. Chin J Cancer Res. (2019) 31:825. doi: 10.21147/j.issn.1000-9604.2019.05.12 31814686 PMC6856700

[7] Li X Huang C Xie X Wu Z Tian X Wu Y. The impact of smoking status on the progression-free survival of non-small cell lung cancer patients receiving molecularly target therapy or immunotherapy versus chemotherapy: a meta-analysis. J Clin Pharm Ther. (2021) 46:256–66. doi: 10.1111/jcpt.13309, PMID: 33152129

[8] Makadzange EE Peeters A Joore MA Kimman ML. The effectiveness of health education interventions on cervical cancer prevention in Africa: a systematic review. Prev Med. (2022) 164:107219. doi: 10.1016/j.ypmed.2022.107219 36007752

[9] Xu L Odum M. Cancer awareness and behavioral determinants associated with cancer prevention—a quantitative study among young adults in rural settings. J Cancer Educ. (2019) 34:562–70. doi: 10.1007/s13187-018-1342-8, PMID: 29508230

[10] Al-Azri M Al-Saadi WI Al-Harrasi A Panchatcharam SM. Knowledge of cancer risk factors, symptoms, and barriers to seeking medical help among Omani adolescents. Asian Pacific J cancer prevention: APJCP. (2019) 20:3655. doi: 10.31557/APJCP.2019.20.12.3655 PMC7173372 31870107

[11] Morales-Campos DY Markham CM Peskin MF Fernandez ME. Hispanic mothers' and high school girls' perceptions of cervical cancer, human papilloma virus, and the human papilloma virus vaccine. J Adolesc Health. (2013) 52:S69–75. doi: 10.1016/j.jadohealth.2012.09.020, PMID: 23601613

[12] Barros A Moreira L Santos H Ribeiro N Carvalho L Santos-Silva F. “Cancer–educate to prevent”–high-school teachers, the new promoters of cancer prevention education campaigns. PLoS One. (2014) 9:e96672. doi: 10.1371/journal.pone.0096672 24817168 PMC4016009

[13] Kang J Ciecierski CC Malin EL Carroll AJ Gidea M Craft LL. A latent class analysis of cancer risk behaviors among US college students. Prev Med. (2014) 64:121–5. doi: 10.1016/j.ypmed.2014.03.023, PMID: 24704131 PMC4089896

[14] Kyle RG Forbat L Rauchhaus P Hubbard G. Increased cancer awareness among British adolescents after a school-based educational intervention: a controlled before-and-after study with 6-month follow-up. BMC Public Health. (2013) 13:1–11. doi: 10.1186/1471-2458-13-190 23496855 PMC3599322

[15] Luszczynska A Schwarzer R. Social cognitive theory. Fac Health Sci Publ. (2015) 2015:225–51.

[16] Mukherjee S Kumar U. Psychological resilience: a conceptual review of theory and research. Routledge Int handbook of psychosocial resilience. (2016) 1:3–12. doi: 10.4324/9781315666716

[17] Vella SLC Pai NB. A theoretical review of psychological resilience: defining resilience and resilience research over the decades. Archives Med Health Sci. (2019) 7:233–9. doi: 10.4103/amhs.amhs_119_19

[18] Kagawa Singer M Valdez Dadia A Yu MC Surbone A. Cancer, culture, and health disparities: time to chart a new course? CA Cancer J Clin. (2010) 60:12–39. doi: 10.3322/caac.20051, PMID: 20097836

[19] Olufemi OO Omowumni SR Ajoke OA Olufemi AE. Knowledge and awareness of breast Cancer and screening methods among female undergraduate students in a semi-Urban College of culture and humanities, Nigeria. Int J Caring Sci. (2017) 10:1–12.

[20] Smith WT Tattersall MHN Irwig LM Langlands AO. Undergraduate education about cancer. European J Cancer Clin Oncol. (1991) 27:1448–53. doi: 10.1016/0277-5379(91)90029-D 1720635

[21] Ibrahim AM Fathi Zaghamir DE. Enhancing testicular Cancer prevention among university students: a health belief model and social support intervention. Asian Pac J Cancer Prev. (2024) 25:609–16. doi: 10.31557/APJCP.2024.25.2.609, PMID: 38415548 PMC11077125

[22] Masso-Calderón AM Meneses-Echávez JF Correa-Bautista JE Tovar-Cifuentes A Alba-Ramírez PA Charry-Ángel CE. Effects of an educational intervention on breast self-examination, breast cancer prevention-related knowledge, and healthy lifestyles in scholars from a low-income area in Bogota, Colombia. J Cancer Educ. (2018) 33:673–9. doi: 10.1007/s13187-016-1133-z, PMID: 27815813 PMC5949133

[23] Peacey V Steptoe A Davídsdóttir S Baban A Wardle J. Low levels of breast cancer risk awareness in young women: an international survey. Eur J Cancer. (2006) 42:2585–9. doi: 10.1016/j.ejca.2006.03.017 PMID: 16829071

[24] Heuckmann B Ammann M Asshoff R. Identifying predictors of teachers’ intention and willingness to teach about cancer by using direct and belief-based measures in the context of the theory of planned behaviour. Int J Sci Educ. (2020) 42:547–75. doi: 10.1080/09500693.2020.1717671

[25] Sanidas EE Aggelaki S Xomeritaki H Godikakis E Tsiftsis DD. The influence of undergraduate medical cancer education on students’ sensitivity towards cancer. J Cancer Educ. (1993) 8:19–23. PMID: 8489905 10.1080/08858199309528202

[26] Sarkar U Le GM Lyles CR Ramo D Linos E Bibbins-Domingo K. Using social media to target cancer prevention in young adults. J Med Internet Res. (2018) 20:e203. doi: 10.2196/jmir.8882 29871850 PMC6008512

[27] Davis R Loescher LJ Rogers J Spartonos D Snyder A Koch S. Evaluation of project students are Sun safe (SASS): a university student-delivered skin cancer prevention program for schools. J Cancer Educ. (2015) 30:736–42. doi: 10.1007/s13187-014-0742-7, PMID: 25417824

[28] Girschik J Miller LJ Addiscott T Daube M Katris P Ransom D. Precision in setting cancer prevention priorities: synthesis of data, literature, and expert opinion. Front Public Health. (2017) 5:125. doi: 10.3389/fpubh.2017.00125 28634579 PMC5459884

[29] Spring B King AC Pagoto SL Van Horn L Fisher JD. Fostering multiple healthy lifestyle behaviors for primary prevention of cancer. Am Psychol. (2015) 70:75. doi: 10.1037/a0038806 25730716 PMC4626078

[30] Lee YA. Evaluating knowledge, attitudes and health behavior regarding cancer among college students based on 10 national cancer prevention recommendations. Korean J Emergency Medical Services. (2019) 23:125–38.

[31] DeRosier ME Frank E Schwartz V Leary KA. The potential role of resilience education for preventing mental health problems for college students. Psychiatr Ann. (2013) 43:538–44. doi: 10.3928/00485713-20131206-05

[32] Pedrelli P Nyer M Yeung A Zulauf C Wilens T. College students: mental health problems and treatment considerations. Acad Psychiatry. (2015) 39:503–11. doi: 10.1007/s40596-014-0205-9 25142250 PMC4527955

[33] Uygun E. The relation between Syrians’ quality of life, depression and anxiety levels and economic conditions: a cross-sectional study at an adult refugee mental health clinic in Turkey. Anatolian J Psychiatry. (2020) 21:1–11. doi: 10.5455/apd.71190

[34] Eisenberg D Downs MF Golberstein E Zivin K. Stigma and help seeking for mental health among college students. Med Care Res Rev. (2009) 66:522–41. doi: 10.1177/1077558709335173, PMID: 19454625

[35] Ahorsu DK Sánchez Vidaña DI Lipardo D Shah PB Cruz González P Shende S. Effect of a peer-led intervention combining mental health promotion with coping-strategy-based workshops on mental health awareness, help-seeking behavior, and wellbeing among university students in Hong Kong. Int J Ment Heal Syst. (2021) 15:1–10. doi: 10.1186/s13033-020-00432-0 PMC7796456 33422098

[36] Kitzrow MA. The mental health needs of today's college students: challenges and recommendations. NASPA J. (2009) 46:646–60. doi: 10.2202/1949-6605.1310

[37] Salimi N Gere B Talley W Irioogbe B. College students mental health challenges: concerns and considerations in the COVID-19 pandemic. J Coll Stud Psychother. (2023) 37:39–51. doi: 10.1080/87568225.2021.1890298

[38] Auerbach RP Alonso J Axinn WG Cuijpers P Ebert DD Green JG. Mental disorders among college students in the World Health Organization world mental health surveys. Psychol Med. (2016) 46:2955–70. doi: 10.1017/S0033291716001665 PMID: 27484622 PMC5129654

[39] Eisenberg D Hunt J Speer N Zivin K. Mental health service utilization among college students in the United States. J Nerv Ment Dis. (2011) 199:301–8. doi: 10.1097/NMD.0b013e3182175123, PMID: 21543948

[40] Hubbard K Reohr P Tolcher L Downs A. Stress, mental health symptoms, and help-seeking in college students. Psi Chi J Psychol Res. (2018) 23:293–305. doi: 10.24839/2325-7342.JN23.1.2

[41] Sontag-Padilla L Dunbar MS Ye F Kase C Fein R Abelson S. Strengthening college students’ mental health knowledge, awareness, and helping behaviors: the impact of active minds, a peer mental health organization. J Am Acad Child Adolesc Psychiatry. (2018) 57:500–7. doi: 10.1016/j.jaac.2018.03.019, PMID: 29960695

[42] Loo JL Woo WY Chin MW Yam HR Ang YK Yim HS. Cancer awareness of a sample of Malaysian undergraduate students. American J Cancer Prevention. (2013) 1:9–13. doi: 10.12691/ajcp-1-1-3 23534795

[43] Burke AD Burns JW Chakraborty S Saha T Ray A Borsch DM. Evaluation of cancer awareness, cancer education, and prevention intervention techniques among university-level students in the United States and India. J Educ Health Promot. (2022) 11:187. doi: 10.4103/jehp.jehp_1422_21 36003241 PMC9393919

[44] Zhang Y Sang X Wu Y Liu T Niu R Han L. Correlation between frequency of eating out of home and dietary intake, sleep, and physical activity: a survey of young CDC employees in China. Int J Environ Res Public Health. (2022) 19:3209. doi: 10.3390/ijerph19063209 35328895 PMC8953287

[45] Feng LS Wu XQ Li QL Yang Q Yin FL Wang QY. Development and reliability and validity test of the fear of Cancer scale (FOCS). Ann Med. (2022) 54:2353–61. doi: 10.1080/07853890.2022.2113914 PMC9467598 36066037

[46] Hammermeister J Mount SE Jordan C Briggs L Galm R. The relationship between mental fitness skills, psychological resilience, and academic achievement among first generation college students. Coll Stud J. (2020) 54:13–24.

[47] Wu Y Sang ZQ Zhang XC Margraf J. The relationship between resilience and mental health in Chinese college students: a longitudinal cross-lagged analysis. Front Psychol. (2020) 11:450850. doi: 10.3389/fpsyg.2020.00108 PMC7012791 32116918

[48] Biddle SJ Asare M. Physical activity and mental health in children and adolescents: a review of reviews. Br J Sports Med. (2011) 45:886–95. doi: 10.1136/bjsports-2011-090185, PMID: 21807669

[49] Hartley MT. Investigating the relationship of resilience to academic persistence in college students with mental health issues. Rehabil Couns Bull. (2013) 56:240–50. doi: 10.1177/0034355213480527

[50] Wu E Tiggelaar SM Jiang T Zhao H Wu R Wu R. Cervical cancer prevention-related knowledge and attitudes among female undergraduate students from different ethnic groups within China, a survey-based study. Women Health. (2018) 58:661–84. doi: 10.1080/03630242.2017.1333076, PMID: 28532334

[51] Abraham C Sheeran P. The health belief model. Predicting health behaviour: Res practice with social cognition models. (2015) 2:30–55.

[52] Skinner CS Tiro J Champion VL. Background on the health belief model. Health Behav Theory, Res Prac. (2015) 75:1–34.

[53] Kim HS Ahn J No JK. Applying the health belief model to college students' health behavior. Nutr Res Pract. (2012) 6:551. doi: 10.4162/nrp.2012.6.6.551 23346306 PMC3542446

[54] Guilford K McKinley E Turner L. Breast cancer knowledge, beliefs, and screening behaviors of college women: application of the health belief model. Am J Health Educ. (2017) 48:256–63. doi: 10.1080/19325037.2017.1316694

[55] Nobiling BD Maykrantz SA. Exploring perceptions about and behaviors related to mental illness and mental health service utilization among college students using the health belief model (HBM). Am J Health Educ. (2017) 48:306–19. doi: 10.1080/19325037.2017.1335628

[56] Priest HM Knowlden AP Sharma M. Social cognitive theory predictors of human papillomavirus vaccination intentions of college men at a southeastern university. Int Q Community Health Educ. (2015) 35:371–85. doi: 10.1177/0272684X15583289, PMID: 26470399

[57] Zhang W. A study analyzing the intention of HPV vaccination among Chinese female college students based on social cognitive theory. J Educ Human Soc Sci. (2024) 28:619–27. doi: 10.54097/59186573

[58] Ishibashi A Okamura J Ueda R Sunami S Kobayashi R Ogawa J. Psychosocial strength enhancing resilience in adolescents and young adults with cancer. J Pediatr Oncol Nurs. (2016) 33:45–54. doi: 10.1177/1043454214563935, PMID: 25862715

[59] Macía P Gorbeña S Barranco M Alonso E Iraurgi I. Role of resilience and emotional control in relation to mental health in people with cancer. J Health Psychol. (2022) 27:211–22. doi: 10.1177/1359105320946358 32783467 PMC8739566

[60] Madhukumar S Thambiran UR Basavaraju B Bedadala MR. A study on awareness about breast carcinoma and practice of breast self-examination among basic sciences’ college students. Bengaluru J Fam Med Prim Care. (2017) 6:487–90. doi: 10.4103/2249-4863.222026 PMC5787941 29416994

[61] Tang Y Li Y. Parental death, grief and bereavement: a qualitative study of college students who have a parent with cancer. Asian Soc Sci. (2019) 15:56. doi: 10.5539/ass.v15n8p56

[62] Juntasopeepun P Suwan N Phianmongkhol Y Srisomboon J. Factors influencing acceptance of human papillomavirus vaccine among young female college students in Thailand. Int J Gynaecol Obstet. (2012) 118:247–50. doi: 10.1016/j.ijgo.2012.04.015, PMID: 22727336

[63] Niu Z Willoughby JF Mei JLS Hu P. A cross-cultural comparison of an extended planned risk information seeking model on mental health among college students: cross-sectional study. J Med Internet Res. (2020) 22:e15817. doi: 10.2196/15817 32441654 PMC7275260

[64] Ali AM Hori H Kim Y Kunugi H. The depression anxiety stress scale 8-items expresses robust psychometric properties as an ideal shorter version of the depression anxiety stress scale 21 among healthy respondents from three continents. Front Psychol. (2022) 13:799769. doi: 10.3389/fpsyg.2022.799769 35496141 PMC9044488

[65] Osman A Wong JL Bagge CL Freedenthal S Gutierrez PM Lozano G. The depression anxiety stress scales—21 (DASS-21): further examination of dimensions, scale reliability, and correlates. J Clin Psychol. (2012) 68:1322–38. doi: 10.1002/jclp.21908, PMID: 22930477

[66] Feizi A Kazemnejad A Babaee G Parsayekta Z Monjamed Z. Public awareness of risk factors for cancer and its determinants in an Iranian population. Asia Pac J Public Health. (2010) 22:76–88. doi: 10.1177/1010539509350768, PMID: 20032037

[67] Brayley J Stanton LK Jenner L Paul SP. Recognition and management of leukaemia in children. Br J Nurs. (2019) 28:985–92. doi: 10.12968/bjon.2019.28.15.985, PMID: 31393775

[68] Anderson AS Goode JVR. Engaging students in wellness and disease prevention services. Am J Pharm Educ. (2006) 70:1–16. doi: 10.5688/aj700240 17149419 PMC1636924

[69] Cheng C Dong D He J Zhong X Yao S. Psychometric properties of the 10-item Connor-Davidson resilience scale (CD-RISC-10) in Chinese undergraduates and depressive patients. J Affect Disord. (2020) 261:211–20. doi: 10.1016/j.jad.2019.10.018, PMID: 31654919

[70] Searle W Ward C. The prediction of psychological and sociocultural adjustment during cross-cultural transitions. Int J Intercult Relat. (1990) 14:449–64. doi: 10.1016/0147-1767(90)90030-Z

[71] Athey S Imbens GW. Identification and inference in nonlinear difference-in-differences models. Econometrica. (2006) 74:431–97. doi: 10.1111/j.1468-0262.2006.00668.x

[72] Stuart EA Huskamp HA Duckworth K Simmons J Song Z Chernew ME. Using propensity scores in difference-in-differences models to estimate the effects of a policy change. Health Serv Outcome Res Methodol. (2014) 14:166–82. doi: 10.1007/s10742-014-0123-z PMID: 25530705 PMC4267761

[73] Kushi LH Byers T Doyle C Bandera EV McCullough M Gansler T. American Cancer Society guidelines on nutrition and physical activity for cancer prevention: reducing the risk of cancer with healthy food choices and physical activity. CA Cancer J Clin. (2006) 56:254–81. doi: 10.3322/canjclin.56.5.254 PMID: 17005596

[74] Zhang D Lee EK Mak EC Ho CY Wong SY. Mindfulness-based interventions: an overall review. Br Med Bull. (2021) 138:41–57. doi: 10.1093/bmb/ldab005 33884400 PMC8083197

[75] Klufas A Shin G Raphael R Sarfaty SC Hirsch AE. A thorough analysis of the current state of cancer education in medical schools and application of experimental teaching techniques and their efficacy. Adv Med Educ Pract. (2020) 11:931–46. doi: 10.2147/AMEP.S268382 33293885 PMC7719335

[76] Evans RE Simon AE Wardle J. Public perceptions of the harms and benefits of testicular cancer education: a qualitative study. Cancer Epidemiol. (2010) 34:212–9. doi: 10.1016/j.canep.2009.12.008, PMID: 20060797

[77] Lu H Xie J Gerido LH Cheng Y Chen Y Sun L. Information needs of breast cancer patients: theory-generating meta-synthesis. J Med Internet Res. (2020) 22:e17907. doi: 10.2196/17907 32720899 PMC7420822

[78] Aydın R Karakısla FS Kabukcuoğlu K. Determination of the relationship between gynecological Cancer awareness and fear of Cancer in women using social media and the affecting factors. Cancer Nurs. (2023) 10:1097. doi: 10.1097/NCC.0000000000001230 36927704

[79] Dey S Sharma S Mishra A Krishnan S Govil J Dhillon PK. Breast cancer awareness and prevention behavior among women of Delhi, India: identifying barriers to early detection. Breast Cancer: Basic Clin Res. (2016) 10:40358. doi: 10.4137/BCBCR.S40358 PMC5074580 27789957

[80] Denckla CA Cicchetti D Kubzansky LD Seedat S Teicher MH Williams DR. Psychological resilience: an update on definitions, a critical appraisal, and research recommendations. Eur J Psychotraumatol. (2020) 11:1822064. doi: 10.1080/20008198.2020.1822064 33244362 PMC7678676

[81] Flood A Keegan RJ. Cognitive resilience to psychological stress in military personnel. Front Psychol. (2022) 13:809003. doi: 10.3389/fpsyg.2022.809003 35369170 PMC8966027

[82] Zangeneh S Savabi-Esfahani M Taleghani F Sharbafchi MR Salehi M. A silence full of words: sociocultural beliefs behind the sexual health of Iranian women undergoing breast cancer treatment, a qualitative study. Support Care Cancer. (2023) 31:84. doi: 10.1007/s00520-022-07502-8 PMC9792940 36574074

[83] Agha N Rind RD. Beliefs and perceptions about breast cancer among the people living in rural and less privileged areas in Sindh. Pakistan Health Educ. (2021) 121:200–14. doi: 10.1108/HE-10-2020-0101

